# Supplementary figures and images for: Comprehensive analysis of prostate cancer life expectancy, loss of life expectancy, and healthcare expenditures: Taiwan national cohort study spanning 2008 to 2019
Source: PLoS One. 2025 Apr 8;20(4):e0310613. doi: 10.1371/journal.pone.0310613 (PMC11978119; doi:10.1371/journal.pone.0310613)

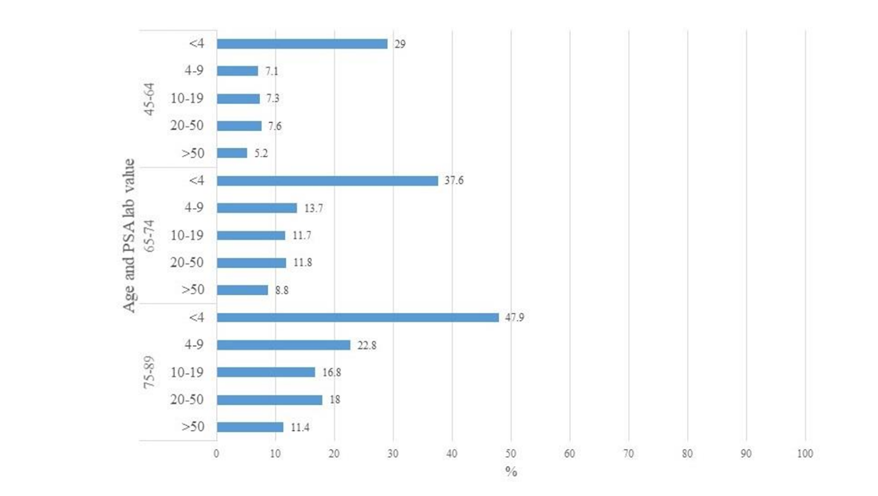

Supplement: S1 Fig — (TIF) [file pone.0310613.s001.tif]
