# Supplementary material for: Comprehensive analysis of prostate cancer life expectancy, loss of life expectancy, and healthcare expenditures: Taiwan national cohort study spanning 2008 to 2019
Source: PLoS One. 2025 Apr 8;20(4):e0310613. doi: 10.1371/journal.pone.0310613 (PMC11978119; doi:10.1371/journal.pone.0310613)
Supplement: Attachment — Submitted filename: Response to Reviewers.docx [file pone.0310613.s003.docx]

PONE-D-24-37580

Author response:

Response to Reviewer #1

Q1: 1. In lines 51-52 the authors mentioned “the healthcare cost of synchronous metastatic PCa in all age groups is higher than the average cost for PCa patients”. But I couldn’t find the data that indicate the average cost for non-synchronous metastatic PCa in the tables. Please present this part of data in an appropriate way.

A1: The annually average cost of prostate cancer was added in line 255.

Q2: 2. I recommend the authors draw figures to better illustrate the noteworthy variations in loss of life expectancy, as previous similar studies (PMID: 37152349) (PMID: 33295139) showed.

A2: The illustrations were added and labeled as figure 1-3, stratified by age PSA level and Gleason score respectively.

Q3: 3. More recent references are required in the manuscript.

A3: Several new references were cited and added at discussion section. (References 19-20)

Responding to Reviewer #2

Q1: In the statement, “The loss of LE in the three groups is 13.63, 6.75, and 3.87 years, respectively,” it is unclear whether this refers to the three risk categories or the three age groups. Please clarify.

A1: It is refers as three age groups: ages 20-64 (13.63 years); ages 65-74 (6.75 years) and ages 75-89 (3.87 years)

Q2: Regarding the sentence, “The healthcare cost of synchronous metastatic PCa in all age groups is higher than the average cost for PCa patients in Taiwan,” I suggest including some brief data to support this statement.

A2: The annually average cost of prostate cancer was added in line 255.

Q3: The staging of PCa is not mentioned. Did you use the D’Amico classification, the NCCN classification, or another system? Additionally, how did you group the patients into the four-stage categories?

A3: This study uses the AJCC cancer staging (8th edition) for staging prostate cancer. The data was collected from Taiwan Cancer Registry database which included prostate cancer stage. The first reason for using four-stage categories is that it is the same as the staging system currently used clinically. Secondly, it facilitates communication and discussion among research members.
